# Coupling Between Leg Muscle Activation and EEG During Normal Walking, Intentional Stops, and Freezing of Gait in Parkinson's Disease

**DOI:** 10.3389/fphys.2019.00870

**Published:** 2019-07-12

**Authors:** Moritz Günther, Ronny P. Bartsch, Yael Miron-Shahar, Sharon Hassin-Baer, Rivka Inzelberg, Jürgen Kurths, Meir Plotnik, Jan W. Kantelhardt

**Affiliations:** ^1^Institute of Physics, Martin-Luther-University Halle-Wittenberg, Halle, Germany; ^2^Department of Physics, Bar-Ilan University, Ramat Gan, Israel; ^3^Center of Advanced Technologies in Rehabilitation, Sheba Medical Center, Tel Hashomer, Israel; ^4^Neuroscience Department, Sackler Faculty of Medicine, School of Graduate Studies, Tel-Aviv University, Tel Aviv, Israel; ^5^Sagol Neuroscience Center and Department of Neurology, Sheba Medical Center, Movement Disorders Institute, Tel-Hashomer, Israel; ^6^Sackler Faculty of Medicine, Tel-Aviv University, Tel Aviv, Israel; ^7^Department of Neurology and Neurosurgery, Sackler Faculty of Medicine, Tel-Aviv University, Tel Aviv, Israel; ^8^Sagol School of Neuroscience, Tel Aviv University, Tel Aviv, Israel; ^9^Department of Applied Mathematics and Computer Science, The Weizmann Institute of Science, Rehovot, Israel; ^10^Potsdam Institute for Climate Impact Research, Potsdam, Germany; ^11^Department of Physics, Humboldt University of Berlin, Berlin, Germany; ^12^Saratov State University, Saratov, Russia; ^13^Department of Physiology and Pharmacology, Sackler Faculty of Medicine, Tel-Aviv University, Tel-Aviv, Israel; ^14^Gonda Brain Research Center, Bar Ilan University, Ramat-Gan, Israel

**Keywords:** phase synchronization, non-linear coupling, time series analysis, EMG, EEG, Parkinson's disease, freezing of gait

## Abstract

In this paper, we apply novel techniques for characterizing leg muscle activation patterns via electromyograms (EMGs) and for relating them to changes in electroencephalogram (EEG) activity during gait experiments. Specifically, we investigate changes of leg-muscle EMG amplitudes and EMG frequencies during walking, intentional stops, and unintended freezing-of-gait (FOG) episodes. FOG is a frequent paroxysmal gait disturbance occurring in many patients suffering from Parkinson's disease (PD). We find that EMG amplitudes and frequencies do not change significantly during FOG episodes with respect to walking, while drastic changes occur during intentional stops. Phase synchronization between EMG signals is most pronounced during walking in controls and reduced in PD patients. By analyzing cross-correlations between changes in EMG patterns and brain-wave amplitudes (from EEGs), we find an increase in EEG-EMG coupling at the beginning of stop and FOG episodes. Our results may help to better understand the enigmatic pathophysiology of FOG, to differentiate between FOG events and other gait disturbances, and ultimately to improve diagnostic procedures for patients suffering from PD.

## 1. Introduction

Physiological systems under neural regulation exhibit non-stationary, intermittent, scale-invariant, and non-linear behaviors (Bassingthwaighte et al., 2013), and their dynamics transiently change in time across different physiologic states (Ivanov et al., 1999; Bunde et al., 2000; Kantelhardt et al., 2002; Schmitt et al., 2009; Schumann et al., 2010; Kantelhardt et al., 2015) and under pathologic conditions (Peng et al., 1995; Bartsch et al., 2007a; Hu et al., 2009; Penzel et al., 2016). The structural and neuronal control networks that constitute physiological organ systems lead to a high degree of complexity of their output signals (Ivanov et al., 2009), and this complexity is further increased by various feed-back interactions (Collins et al., 1996; Hegger et al., 1998; Ivanov et al., 1998) and coupling among different systems (Schäfer et al., 1998; Tass et al., 1998; Chen et al., 2006; Bartsch et al., 2007b; Lin et al., 2016; Stramaglia et al., 2016), the nature of which remains not well-understood. Quantifying these physiologic interactions is a challenge as one system can exhibit multiple simultaneous interactions with other systems and organ systems can communicate through several independent and coexisting mechanisms of interaction which operate at different time scales (Bartsch et al., 2012; Bartsch and Ivanov, 2014). To understand physiologic function it is critical to identify physiological interactions and to track their evolution under different physiologic states and pathologic conditions.

In human physiology, interactions have been studied among a variety of different physiological systems. One of the most prominent examples is the coupling between heartbeat and respiration (Schäfer et al., 1998; Bartsch et al., 2007b). Originally described through the periodic variation of the heart rate within a breathing cycle and termed “Respiratory Sinus Arrhythmia” (RSA) (Angelone and Coulter, 1964), it has been shown recently (Bartsch et al., 2014; Penzel et al., 2016) that RSA is only one aspect of cardio-respiratory interaction. Another form of cardiorespiratory coupling can be quantified by cardiorespiratory phase synchronization (Schäfer et al., 1998) which is enhanced under meditation (Cysarz and Büssing, 2005), changes across sleep stages and with healthy aging (Bartsch et al., 2012), and is significantly reduced in subjects after myocardial infarcts (Leder et al., 2000). Interactions between brain dynamics and cardiac activity during sleep are strongest for EEG delta waves (Brandenberger et al., 2001; Jurysta et al., 2003). However, the relative contribution of different brain-wave frequencies to the brain-heart coupling changes for different sleep stages (Bartsch et al., 2015; Faes et al., 2015; Lin et al., 2016). An entrainment between cardiac and locomotor rhythms can be observed during running and cycling (Kirby et al., 1989a), and the onset of cardiolocomotor coupling seems to induce a dissociation of coupling between respiratory and locomotor rhythms as well as reduces cardiorespiratory synchronization (Niizeki et al., 1993). In Parkinson's disease (PD) resting tremor a strong interdependence between the EMG of forearm muscles and activity in the contralateral primary motor cortex has been demonstrated (Tass et al., 1998), which is effective primarily in the single and double tremor frequency range (Timmermann et al., 2003). Recent studies on other involuntary movements and tremor syndromes suggest that different movement disorders could be discriminated by neurophysiological means through cerebro-muscular and cerebro-cerebral coupling analysis (Timmermann et al., 2003; Klimesch, 2018).

In this paper we investigate cerebro-muscular coupling between EEG and EMG activation patterns in lower leg muscles in order to characterize normal gait and to distinguish between intentional stops and unintended freezing-of-gait (FOG) episodes. FOG is a paroxysmal gait disturbance seen in about half of the persons with PD in the more advanced stages of the disease (see Nutt et al., 2011; Snijders et al., 2016 for reviews). During FOG “attacks” the sufferer is unable to generate effective stepping, and rather trembles in place with minimal progression, or simply “freezes” in place. Several studies have documented FOG-associated changes in physiological signals such as electroencephalography (EEG; Shine et al., 2014; Handojoseno et al., 2015; Ly et al., 2017a,b), electrocardiography (Maidan et al., 2010), galvanic skin response (Mazilu et al., 2015), electromyography (EMG; Nieuwboer et al., 2004), and kinematics (Bächlin et al., 2010). These changes are probably associated with cognitive, mental, spinal, motor and autonomic nervous system functions. However, it is not known whether these presumably independent functions are interacting in the context of FOG.

## 2. Materials and Methods

EEG and EMG data were recorded from participants with PD and from elderly controls (EC). Inclusion criteria for PD participants were: age above 50 years, diagnosis of idiopathic PD according to the UK Brain Bank Criteria (Hughes et al., 1992), current levodopa treatment, ability to walk unassisted and without pain for at least 100 m, and being able to understand and perform verbal instructions. Exclusion criteria were the presence of significant co-morbidities and major orthopedic problems. PD participants were examined in the OFF state, i.e., at least 12 h after the last intake of anti-PD medications. The study protocol was approved by the Institutional Review Board (IRB) of the Sheba Medical Center, and the experiments were conducted in the Center of Advanced Technologies in Rehabilitation of the medical center (see Shahar et al., 2019 for a thorough description of the study).

Following the screening for eligibility, 25 participants (17 PD, 8 EC) agreed to participate and gave written informed consent prior to the study. Two PD participants were unable to complete the assessment protocol. Simultaneous (parallel) recordings of 32-channel surface EEG and 4-channel leg-muscle surface EMG were finally obtained from 4 EC and 9 PD participants during figure-eight walking experiments (see below). One EC participant had to be excluded because the EMG recording consisted mainly of artifacts. The group of PD patients was further divided into those that showed the FOG symptom during the experiments (PD+FOG, 4 participants) and those who did not show FOG (PD-FOG, 5 participants). Among the former group 71 FOG episodes were observed (17.8 ± 8.1 per participant). The demographic and clinical data of the study cohorts are summarized in Table 1. We did not differentiate between males and females because of the small sample size and because a possible gender effect on the phenomenology of FOG has not been reported. Regarding FOG prevalence, a large cohort of 490 PD participants found similar percentages for FOG in men and women (Szewczyk-Krolikowski et al., 2014).

**Table 1:** Demographic and clinical data of the study cohorts: Elderly controls (EC), participants with PD that do not show freezing-of-gait (PD−FOG), and participants with PD and freezing-of-gait (PD+FOG).

**Group**	**EC**	**PD-FOG**	**PD+FOG**
f/m	1/2	2/3	0/4
Age [y]	65.7 ± 14.2	68.6 ± 9.0	64.3 ± 8.2
BMI [kg/m^2^]	23.5 ± 3.3	24.7 ± 2.9	26.6 ± 6.7
MoCA	24.0 ± 2.6	24.2 ± 5.9	22.8 ± 2.2
UPDRS		10.8 ± 4.9	12.8 ± 5.6
Number of stops	13	19	22
Number of FOG			62

*Gender f/m, females/males; BMI, body mass index (in kg/m^2^); MoCA, Montreal Cognitive Assessment (Lifshitz et al., 2012); UPDRS, Unified PD Rating Scale, Score Part III (Fahn et al., 1987). Differences between the groups are not significant. All participants performed multiple trials of the experiment; the listed total numbers of stop and FOG episodes was used for the analysis*.

All participants performed gait trials during which they were exposed to “FOG triggers,” i.e., gait tasks that are highly probable to invoke FOG episodes among affected persons (figure-eight shaped walking trajectory and narrow passage Plotnik et al., 2014). Occasionally the participants were instructed to stop walking (i.e., “commanded stops”) to provide a controlled condition in contrast to the unintended FOG episodes. In addition, a 30-s recording was obtained during continuous standing still. EEG activity was recorded by a portable system (Micromed, Mogliano Veneto, Italy) consisting of a 32-channel montage using the international 10–20 electrode placement scheme. In addition, four surface EMG channels (tibialis anterior and gastrocnemius muscles of each leg) were recorded simultaneously by the same device. The data were annotated by *post-hoc* analysis of video files recorded during the gait trials. Data slices were sorted according to motion type (walking, freezing, commanded stops).

### 2.1. Signal Processing

EEG data were pre-processed using the EEGLAB software (Delorme et al., 2011). For each gait task and each participant, data pre-processing steps included: (i) omitting data from electrodes with high impedance (>10*kΩ*) and high standard deviation; (ii) data down-sampling from 2,048 to 256 Hz; (iii) a basic finite impulse response high-pass filtering with a threshold of 0.1 Hz; (iv) applying an Independent Component Analysis (ICA) (Bell and Sejnowski, 1995) (“runica” implementation) for the removal of eye movements and general movement artifacts. The ICA algorithm exploits the fact that several EEG electrodes are affected by the same artifacts, in particular movement artifacts. This common “source” is identified by the algorithm, and its relative contribution to each electrode is subtracted. Using component activation, spectra and maps, the different components were visually inspected, and a minimal number of components (2 or 3) was removed. A similar ICA approach has previously been used for removing EEG movement artifacts during walking (see, e.g., Gwin et al., 2011; Arad et al., 2018).

**Figure 1:**
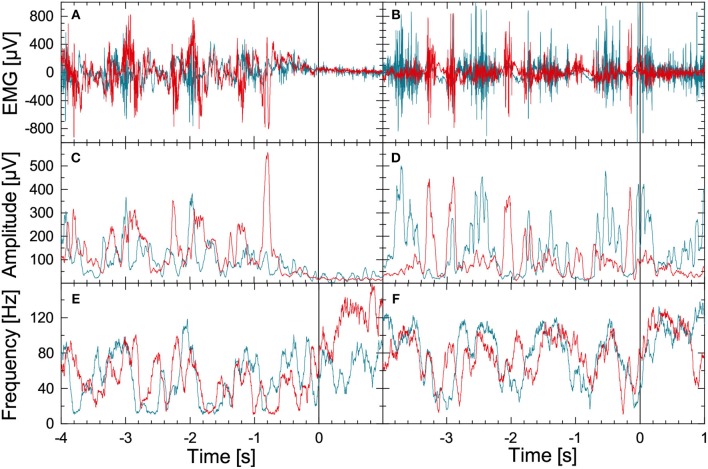
(Color online) Illustration of EMG data preprocessing as optimized for the data recorded during FOG-provoking experiments. **(A)** Raw EMG signals recorded with electrode pairs for the right musculus gastrocnemius (blue) and the right musculus tibialis anterior (red) in a PD subject during normal walking (time: –4 to 0 s) and during a commanded stop (time: 0 to +1 s). A Hilbert transform and the analytic signal approach were applied to these data to derive **(B)** instantaneous amplitude, and **(C)** instantaneous frequency series. **(D–F)** Raw EMG and corresponding instantaneous amplitude and frequency series for a PD subject during walking (time: –4 to 0 s) and during FOG (time: 0 to +1 s). Note the significant difference in EMG between the stop and the FOG episode.

Figure 1 illustrates the methodology for the leg-muscle surface EMG signal analysis. We begin with the raw EMG data from the right musculus gastrocnemius (blue) and the right musculus tibialis anterior (red) during normal walking (seconds –4 to 0) followed by a commanded stop (seconds 0 to +1; top left panel) or FOG episode (seconds 0 to +1; top right panel). Data for both parts were recorded during a figure-eight walking experiment. The EMG records of the corresponding left muscles show a similar behavior but are not displayed for the sake of clarity. The instantaneous amplitudes and instantaneous frequencies are derived in several processing steps, see Figures 1B,C, respectively. Firstly, a FFT high-pass filter (Theiler et al., 1992) with a limit frequency at 10 Hz is applied to the raw EMG signal (sampled at 2048 Hz) to eliminate DC components and artifacts, e.g., due to electrode motion during gait. The resulting detrended signal *x*^(*j*)^(*t*) oscillates at varying amplitudes with frequencies between 10 and up to 200 Hz for each of the four EMG recordings (j=1,…, 4, two muscles and two legs). A Hilbert transform (Gabor, 1946; Boashash, 1992) of *x*^(*j*)^(*t*) yields the analytic signal

(1) $$\begin{array}{l} x^{\left(j\right)}\left(t\right)+i{\tilde{x}}^{\left(j\right)}\left(t\right)=A^{\left(j\right)}\left(t\right)\exp\left(i\varphi^{\left(j\right)}\left(t\right)\right), \end{array}$$

(*i* = imaginary unit) which is used to reconstruct instantaneous amplitudes *A*^(*j*)^(*t*) and instantaneous phases φ^(*j*)^(*t*) of oscillations that are related to the activation of several lower leg muscle fibers. Instantaneous frequencies $f^{\left(j\right)}\left(t\right)=\frac{1}{2\pi }\left[\varphi^{\left(j\right)}\left(t\right)-\varphi^{\left(j\right)}\left(t-\Delta t\right)\right]/\Delta t$ are defined accordingly. In practice, we use discrete time steps, and $A_{k}^{\left(j\right)}=A^{\left(j\right)}\left(k\Delta t\right)$. The Hilbert transformed ${\tilde{x}}_{k}$ can be calculated by FFT: (i) transforming *x*_*k*_ into Fourier space, (ii) multiplying all Fourier coefficients ω_*k*_ by −*i*sgn(ω_*k*_), and (iii) applying the inverse Fourier transform Rosenblum et al. (1996). Finally, moving average filters with window lengths of 0.05 and 0.1 s have been applied to the amplitude data (Figures 1B,E) and to the frequency data (Figures 1C,F). While the stepping pattern is visible for *t* < 0 in all figures, there is a drastic drop in EMG amplitudes and a certain rise in EMG frequencies (i.e., for the musculus tibialis anterior) after the stop command (Figure 1, left hand side). In contrast, during FOG there is no significant change in EMG amplitudes and frequencies (Figure 1, right hand side). The EMG-based amplitude time series *A*^(*j*)^(*t*) and the frequency time series *f*^(*j*)^(*t*) were resampled to 8 Hz for further analysis.

## 3. Results

**Figure 2:**
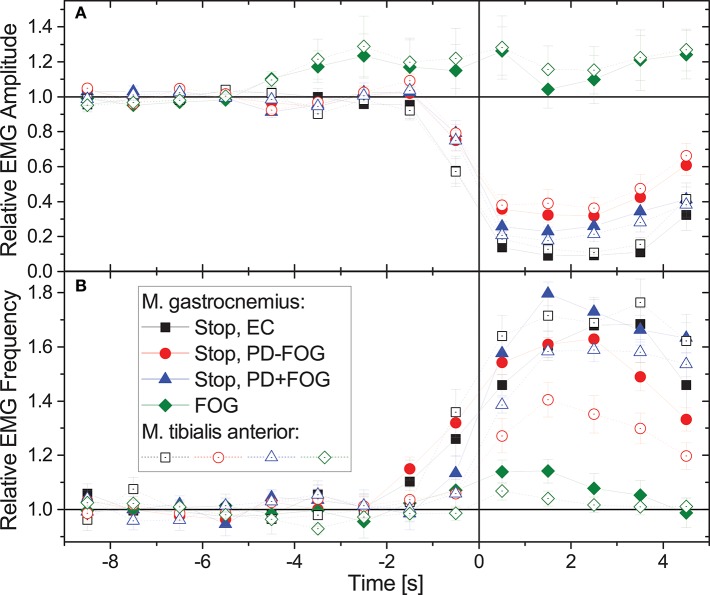
(Color online) Relative changes of **(A)** EMG amplitudes and **(B)** EMG frequencies during gait (time: –9 to 0 s) followed by a commanded stop or a FOG event (time: 0 to +5 s). Time 0 (the vertical line) corresponds to a complete stop or FOG event (according to video footage). In case of stopping the command to stop was given 1–2 s earlier, which explains the changes starting before 0. Data have been normalized to 1 for normal walking (time from –9 to –5 s). For stops, data from the three groups show a similar behavior, i.e., significant drops in EMG amplitude and increases in EMG frequency. However, for FOG events EMG amplitudes and frequencies hardly change. Full symbols are for musculus gastrocnemius and open symbols for musculus tibialis anterior (no significant differences); data from left and right leg and all available episodes and subjects have been averaged. Error bars indicate the standard error of the mean (standard deviation divided by square root of number of stop/FOG epochs). We note that no points that appear to the left of the vertical line (time 0) are affected by data measured after time 0. This also holds vice versa, except for the normalization coming for the data measured more than 5 s before time 0.

Figure 2 compares the average behavior of EMG amplitudes and EMG frequencies during normal gait and commanded stops in the three groups, EC, PD-FOG, and PD+FOG as well as during FOG events (PD+FOG only). Data have been normalized to 1 for normal walking (time from −9 to −5 s), and averaging has been applied to all corresponding events in figure-eight and narrow-passage gait experiments; data from the left and the right leg have been averaged as well. While during stops there is a significant drop in EMG amplitudes and a well-pronounced increase in EMG frequencies in all three groups, EMG amplitudes and frequencies do hardly change for FOG events. Student's *t*-tests yield *p* < 0.001 for the deviation of the relative EMG amplitudes from 1 in each subject group, each muscle, and each second (*t* = 0.5 to 4.5 s) after the beginning of the stop episode. For FOG episodes, no significant change is seen (*p*> 0.05 in each case). We observe a slight increase in EMG amplitudes about 4–5 s prior to FOG events (*p* = 0.013 at *t* = −4.5 s, but insignificant for other times) and no further increase or decrease during the actual FOG events. In contrast, EMG frequencies show a slight increase just at the beginning of FOG events (*p* < 0.001 at *t* = +0.5 s and −0.5 s and *p* = 0.05 at *t* = +1.5 s in musculus gastrocnemius, *p* = 0.05 at *t* = +0.5 s in musculus tibialis anterior, insignificant for other times). We note, however, that given the within-subject and inter-subject variations of these changes (as indicated by the error bars in Figure 2), these slight increases in EMG amplitudes and frequencies cannot be used for a reliable detection or even prediction of FOG events.

As shown in Figures 1, 2, EMG amplitudes and frequencies have different average values during normal gait and when standing still. Similar differences occur for the inter-relations between these EMG-derived time series. In order to automatically distinguish between stepping and standing still, we have applied a phase synchronization analysis to all amplitude time series *A*^(*j*)^(*t*) and frequency time series *f*^(*j*)^(*t*) (*j* = 1, …, 4, two muscles and two legs). For each window of 4 s, i. e., each segment of 32 amplitude (or frequency) data points sampled at 8 Hz, we first subtract the local mean and use another Hilbert transform (analogous to Equation 1) to obtain the instantaneous phases of the amplitudes ($\varphi_{A/f}^{\left(j\right)}$, *j* = 1, …, 4) and frequencies ($\varphi_{A/f}^{\left(j\right)}$, *j* = 5, …, 8), respectively (Gans et al., 2009). Then, for each pair of time series (*j, k* = 1, …, 8, *j* ≠ *k*), we calculate the phase synchronization indices γ_*j,k*_ by averaging complex exponentials and taking the absolute value,

(2) $$\begin{array}{l} \gamma_{j,k}\left(t_{0}\right)=|\frac{1}{32}\overset{31/\left(8\text{Hz}\right)}{\underset{t=0}{\sum }}\exp\left[i\varphi_{A/f}^{\left(j\right)}\left(t_{0}+t\right)-i\varphi_{A/f}^{\left(k\right)}\left(t_{0}+t\right)\right]|. \end{array}$$

**Figure 3:**
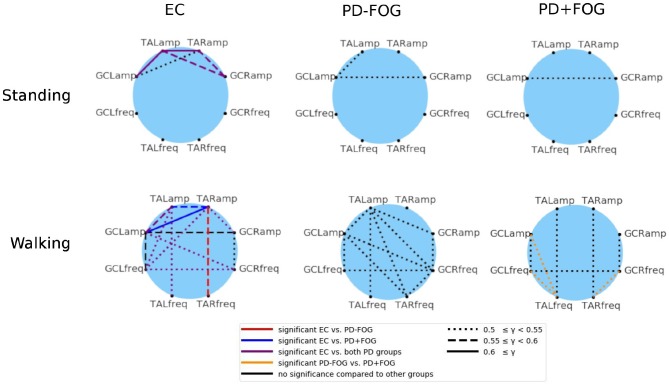
(Color online) Network representation of phase synchronization between EMG-derived amplitudes (four nodes in the top half of each circle, both legs and both muscles) and EMG-derived frequencies (four nodes in the bottom half of each circle). A link is plotted if the corresponding average value of the synchronization index γ (see Equation 2) exceeds an *ad-hoc* limit of 0.5. The link strength is indicated by different line styles (see figure legend): (i) dotted line for 0.5 ≤ γ_*j,k*_ < 0.55, (ii) dashed line for 0.55 ≤ γ_*j,k*_ < 0.6, and (iii) solid line for γ_*j,k*_ ≥ 0.6. The six network plots show group averages for EC (left), PD-FOG (center), and PD+FOG (right) groups; the top panel is for a standing still trial and the bottom panel for figure-eight walking. It is interesting to note that for both PD groups during still standing there is a coupling between EMG amplitudes of GC which is absent in EC subjects. During walking, the amplitude-frequency synchronization increases for all groups, and is most pronounced for EC and much smaller for PD+FOG. Also, PD+FOG subjects show very weak synchronization between right and left leg. Statistically significant links are marked by the following colors (figure legend): (i) in the EC networks - red = links that are significant only for the EC to PD-FOG comparison, dark blue = links that are significant only for the EC to PD+FOG comparison, violet = links that are significant for both comparisons; (ii) in the PD+FOG networks: orange = links that are significant for the PD-FOG to PD+FOG comparison. Significance was probed performing Student's *t*-tests and the significance level was set to *p* < 0.05. TA, musculus tibialis anterior; GC, musculus gastrocnemius; R, right leg; L, left leg; amp, EMG amplitude signal; freq, EMG frequency signal.

Finally, the phase synchronization indices γ_*j,k*_(*t*_0_) are averaged over the whole durations of the experiments, which are either walking in a figure-eight pattern or standing still. Figure 3 shows the group average results for EC, PD-FOG, and PD+FOG groups for standing still (top panel) and figure-eight walking (bottom). Links between a pair of nodes (*j, k*) (representing signals *j* and *k*) appear, if the average value of the corresponding average synchronization index γ_*j,k*_ is larger than an *ad-hoc* limit of 0.5, indicating a pronounced phase synchronization. During still standing (top), only a few links appear for the EC group, and these links are only due to EMG amplitude coupling. In contrast, both PD groups do not show a pronounced phase synchronization during still standing. The picture changes when, for the walking condition (bottom), more links appear in all three groups, and also frequency-frequency as well as amplitude-frequency couplings emerge in addition to amplitude-amplitude coupling. The large number of links in the network of the healthy elderly subjects during walking (bottom left) indicates that a high level of coordination among lower leg muscles is needed to generate normal gait.

**Figure 4:**
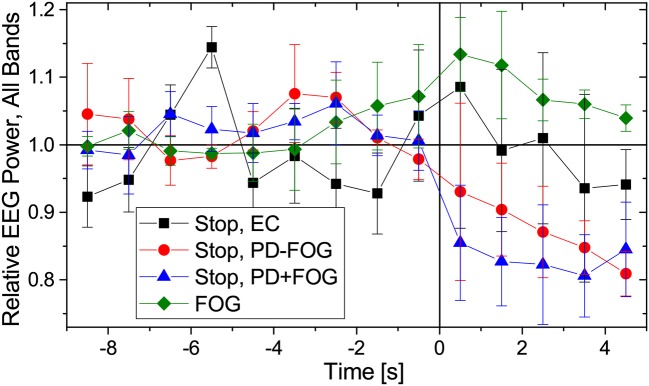
(Color online) Relative changes of EEG amplitudes during normal walking (time: −9 to 0 s) followed by a commanded stop or FOG event (time: 0 to +5 s). As in Figure 2, data have been normalized to 1 for normal walking (time from −9 to −5 s). We have considered the following EEG frequency bands: theta (4–7.5 Hz), alpha (7.5–13 Hz), low beta (13–21 Hz), and high beta (21–35 Hz). Since we found no difference in how EEG amplitudes change for the respective frequencies, we present here an average over all four bands. EEG data from the central electrodes (C3 and C4 closest to the motor cortex) were used; results for other EEG electrodes are similar. Error bars indicate the standard deviations of the relative EEG powers across the four considered bands. As in Figure 2, points to the left of the vertical line are not affected by data recorded after time 0.

Previous work by another group (Shine et al., 2014; Handojoseno et al., 2015) suggests that FOG events can be identified and predicted from EEG signals. In Figure 4 we present changes in EEG amplitudes during normal walking, commanded stops and FOG for the same groups of subjects as in Figure 2. As for the reconstruction of EMG amplitudes, Fourier filtering and a Hilbert transform have been used (see Figure 4 caption for definitions of the EEG frequency bands). Slight decays of EEG amplitudes during commanded stops are visible (for the beta band: Student's *t*-tests *p* < 0.01 from *t* = +0.5 to +4.5 s in PD+FOG and from *t* = +2.5 to +4.5 s in PD-FOG, generally weaker levels of significance for the higher bands, no significant changes in EC subjects), but no significant changes in the EEG amplitudes occur during FOG episodes except for *p* < 0.02 directly at FOG onset (*t* = −0.5 and +0.5 s) in the theta and alpha bands. There may be a slight increase in the EEG amplitude several seconds prior to and during FOG episodes as previously reported (Shine et al., 2014); we obtain *p* = 0.04 at *t* = −3.5 and −2.5 s for the theta band as well as *p* = 0.02 at *t* = −4.5 s in the alpha band and at *t* = −2.5 s in the low beta band; all others are insignificant. However, given the high degree of inter-subject variation, the small number of subjects, and the different types of FOG episodes (turn hesitation, hesitation in tight quarters, open space hesitation, etc.) in our recordings, these changes seem too weak to predict FOG episodes from our limited EEG data. A figure showing the EEG amplitude behavior in each band separately is included in the supplementary material (Figure S2).

**Figure 5:**
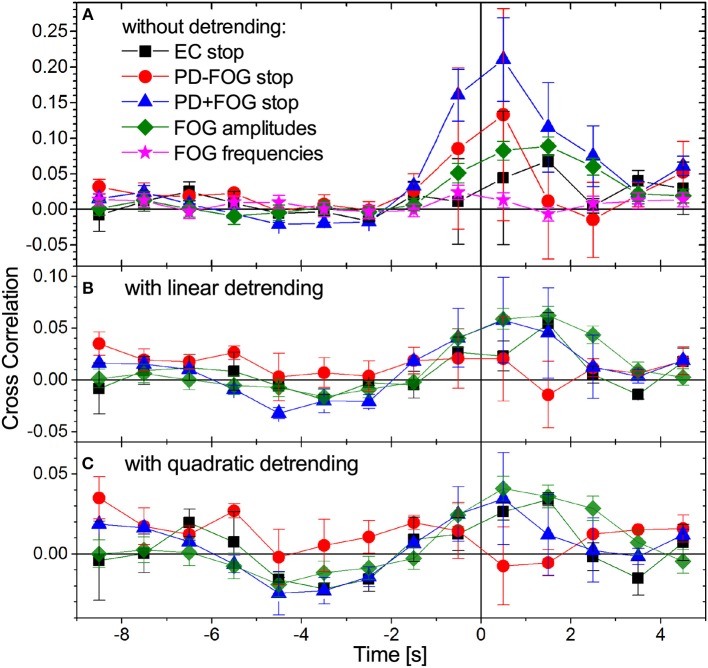
(Color online) Time-dependent cross-correlations between EMG amplitudes and EEG amplitudes during gait (time: –9 to 0 s) followed by a commanded stop or a FOG episode (time: 0 to +5 s). Data for all pairs of EMG amplitude signals (both legs and both muscles) and all EEG amplitude signals (four bands, see caption of Figure 4) have been averaged. The legend in **(A)** is valid for all panels showing results for stop episodes in all three groups of subjects and for FOG episodes in the PD+FOG group, **(A)** without detrending, **(B)** with linear detrending, and **(C)** with quadratic detrending (see Equation 3). The large drop in the cross-correlation curves for stop episodes after detrending (especially for PD+FOG) indicates that the pronounced maximum at the beginning of the stops is mainly caused by non-stationarities (nearly step-like trends) in the amplitude signals. However, the detrended cross-correlation curves for stop and FOG episodes in the PD+FOG group are not clearly different, since there is an overlap of their error bars at all time points. Error bars indicate standard errors of the means. We note that because we are considering 4-s windows in the cross correlation analysis, Equation (3), two data points appearing to the left (right) of the vertical line (time 0) are influenced by data originating from the right (left) of this line. Cross-correlations between EEG amplitudes and EMG frequencies do not change with transition to FOG as also shown in **(A)**.

An important aspect in better understanding the pathophysiology of PD and in particular FOG is the study of inter-relations between different physiological signals, e.g., EEG and EMG. Figure 5 shows cross-correlations between EMG amplitudes (and frequencies) and EEG amplitudes. Since FOG and stop episodes typically last for several seconds, begin (and end) with an uncertainty of approximately 0.5 to 1 s, and our instantaneous EMG and EEG amplitudes have a resolution of 8 Hz (i.e., 8 value per second), we have determined these correlations in 1-s steps, using data from ±2 s (i.e., 32 data points) around each window center. A few seconds of data are needed to statistically distinguish trends associated with the beginning of stop (or FOG) episodes from random fluctuations. Since most of the analyzed signals contain such trends (see Figures 2, 4), we have generalized the standard cross correlation function

(3) $$\begin{array}{l} CC_{j,k}\left(t_{0}\right)=\frac{1}{32SD_{A^{\left(j\right)}}SD_{A^{\left(k\right)}}}\sum_{t=0}^{31/\left(8\text{Hz}\right)}\{\left[A^{\left(j\right)}\left(t_{0}+t\right)-\text{trend}A^{\left(j\right)}\right]\\ \;\times \left[A^{\left(k\right)}\left(t_{0}+t\right)-\text{trend}A^{\left(k\right)}\right]\} \end{array}$$

by subtracting not only the average, $\text{trend}A^{\left(j\right)}=\langle A^{\left(j\right)}\rangle =\frac{1}{32}\overset{31/\left(8\text{Hz}\right)}{\underset{t=0}{\sum }}A^{\left(j\right)}\left(t_{0}+t\right)$ (order 0), but alternatively subtracting a linear (order 1) or quadratic (order 2) regression to fit the (temporarily local) trend of the signal in each 4-s window. Figure 5 compares results for the three groups of subjects and both detrending orders before and during commanded stops and FOG. One can see that linear detrending significantly reduces the EEG-EMG cross-correlations; for PD+FOG as well as PD-FOG this reduction is close to 75%. With second order detrending, cross-correlations even drop below 0.035 in all three groups. This pronounced decline indicates that the cross-correlations between EEG and EMG amplitudes at the beginning of stop episodes are to a large extent due to trends in the original signals (cp. Figures 2, 4). In contrast, for the EEG and EMG amplitude data before and during FOG episodes, cross-correlations are much less affected by detrending and remain > 0.04 even after second order detrending. However, after detrending there is no clear difference between the cross-correlation curves for stops and FOG. The remaining slight increase in detrended EEG-EMG cross-correlation curves for both PD+FOG stop and FOG shortly prior to and during stop/FOG episodes could be due to fast fluctuations that simultaneously occur in both signals. The results are consistent for all considered four EEG bands, several EEG electrodes (most pronounced for central electrodes) and all EMG signals recorded from the four muscles. No cross-correlations occur for EMG frequencies (star symbols in Figure 5A).

## 4. Discussion

### 4.1. Summary of Main Findings

We examined amplitude and frequency characteristics of leg-muscle EMGs and EEGs as well as their coupling during normal walking, voluntary stops and FOG episodes in patients with PD and healthy elderly controls. The pure EMG amplitude and frequency analysis exhibits a characteristic pattern for the transition from normal walking to stopping, i.e., a pronounced decrease in amplitude and an increase in frequency. In contrast, only weak and non-significant changes in EMG amplitudes and frequencies occur at transitions from normal walking to FOG. Clearly, these changes cannot be used for a reliable detection or even prediction of FOG events, although they may help in distinguishing FOG events from normal stops.

There are only a few studies that have examined how FOG is expressed in leg EMG signals. Ynagisawa et al. (1991) described “unique but not uniform patterns of EMG” in five PD+FOG participants suggesting that rhythmic contraction of leg muscles beyond a certain rate is a factor in causing FOG. About a decade later, in a more elaborated study, a consistent pattern of premature timing of Tibialis Anterior and the Gastrocnemius activity was observed before freezing, which was accompanied by a reduction in EMG magnitude, however, data on EMG activity during the freezing episode was not presented (Nieuwboer et al., 2004). Our findings (Figure 2) are in agreement with those shown by Mazzetta et al. (2019), where clear patterns of EMG activities are seen during FOG episodes. Leg EMG activity was associated with freezing since many FOG events involve trembling in place (Schaafsma et al., 2003), or knee trembling which was also implicated with impaired postural adjustments associated with FOG (Jacobs et al., 2009). The finding of EMG activation during FOG events was further elaborated by studies using wearable sensors (Moore et al., 2008; Bächlin et al., 2010) that measured lower limb movements during normal gait and FOG episodes.

Studying EEG amplitudes in many electrodes, we also see only non-significant EEG changes before and during FOG events, irrespective of the considered EEG band. Although there may be a slight increase in EEG amplitudes several seconds prior to and during FOG episodes as previously reported (Shine et al., 2014), these changes seem too weak to predict FOG episodes from our EEG data given the high degree of inter-subject and inter-event variations.

By studying phase synchronization of EMG amplitudes and frequencies we could uncover that pronounced synchronization links between pairs of amplitudes as well as several amplitudes and frequencies and even pairs of frequencies occur during walking, while there is only a weak EMG amplitude synchronization during still standing. The degree of EMG synchronization is generally higher in healthy subjects, while the PD+FOG group showed the least and weakest links. This indicates that normal gait in subjects with PD, in particularly in those with PD and FOG, is already less well-synchronized than in controls.

Finally, we analyzed EMG-EEG amplitude cross-correlations and found pronounced correlations at the beginning of stop and FOG episodes, especially for the PD-FOG and PD+FOG groups. The high susceptibility of these cross-correlations to detrending in the case of stops indicates that they are largely caused by trends in this case. On the other hand, cross-correlations during FOG are much less affected by detrending. However, the resulting detrended cross-correlation curves for FOG and stop episodes are not clearly different. The slight increase in the detrended cross-correlation curves shortly prior and during FOG (and/or stop) episodes could be due to increased EMG-EEG coupling during FOG (and/or stop).

### 4.2. Limitations

A clear limitation of our study is the small number of subjects. From the original 25 participants (17 PD, 8 EC) that were recruited, only 9 PD patients and 3 EC subjects completed the full study protocol and provided EMG data of sufficient quality that could be used for our analysis (EEG data could be used in 23 subjects). Nevertheless, since the subjects performed multiple trials, we obtained a good number of walking, stopping, and FOG epochs as listed in Table 1. Additionally, we have probed our results for significance and mention the p values when discussing the corresponding figures. Because of the small sample size we also could not study gender effects. However, gender effects on the phenomenology of FOG are not known.

Another limitation may be the reconstruction of instantaneous EMG amplitudes and frequencies using the analytic signal approach which includes a Hilbert transform of the original signal. Strictly speaking, a Hilbert transform is established for narrow band signals only. However, in the case of EMG signals (unlike for EEGs) it is not possible to define narrow bands in a consistent fashion, since there are no such bands described in the literature. Overall, we find that a Hilbert transform yields an acceptable reconstruction of an analytical signal for broad-band EMGs, since there is usually only one oscillation present at a given time. This behavior is different from EEGs, where multiple “waves” are present simultaneously. The Hilbert transform has the advantage of deriving both, instantaneous amplitudes and instantaneous frequencies in a combined and therefore fully consistent way. This is not possible for “more direct” (e.g., rectification) approaches, where amplitudes and frequencies must be derived using different procedures. Moreover, our approach yields clearly visible stepping patterns in Figure 1, indicating that even broad-band Hilbert transform results in meaningful amplitudes and frequencies related to the gait cycle. In contrast to rectification methods, Hilbert transform is a linear transformation and not susceptible to artificial frequency doubling. For comparison, we have also applied two alternative methods to derive instantaneous EMG amplitudes and frequencies, see Supplementary Material and Figure S1, and for these approaches we obtain similar results as for the Hilbert transform.

## 5. Conclusions

Our results may help to gain clearer understanding of muscle and brain activation during FOG and how it differs from both walking and intentional stopping. Especially the interaction of the motor cortex and leg muscles is subject to characteristic changes during FOG and seems to be most promising for further research. It seems that neither the leg muscle activation patterns nor the interactions between brain activity and leg muscle activation are interrupted or notably disturbed during FOG. Unlike to what happens in voluntary stops, leg muscle EMGs show a continued activation, and muscle activation seems to be even increasingly correlated with brain activity during FOG. Therefore, our findings seem to point into the direction of a FOG origin in the brain. Either pronounced influences of the brain upon leg muscle activation via fast variations or less well coordinated influences of the brain upon them (compared with normal walking) may be origins of FOG events.

This picture seems to be coherent with modified inter-hemisphere EEG synchronization patterns in subjects with PD, which we have demonstrated in previous work (Shahar et al., 2019). Although we cannot confirm that such changes in EEG patterns could be used to reliably predict actual FOG events (as suggested in Handojoseno et al., 2015), there is a clear evidence for excessive bilateral cortical synchronization during locomotion in subjects with PD, and particularly in subjects with PD and FOG. However, given the pronounced inter-subject and inter-event variability of EMG and EEG variations before and during FOG events, we must conclude that more such data must be recorded and studied before final conclusions on the pathophysiology of the enigmatic FOG events could be reached.

## Author Contributions

MG developed and implemented the EMG data processing methodology, performed the data analysis, prepared the figures, and wrote parts of the paper. RB cosupervised the work of MG, participated in discussions and wrote parts of the paper. YM-S performed all experiments and data recordings and pre-processed the EEG data. SH-B and RI recruited patients and participated in discussions. JK supervised the project and participated in discussions. MP supervised the project and the work of YM-S, participated in discussions, and wrote parts of the paper. JWK devised the data analysis, supervised the work of MG, participated in discussions, and wrote parts of the paper.

### Conflict of Interest Statement

The authors declare that the research was conducted in the absence of any commercial or financial relationships that could be construed as a potential conflict of interest.
