# Supplementary material for: Coupling Between Leg Muscle Activation and EEG During Normal Walking, Intentional Stops, and Freezing of Gait in Parkinson's Disease
Source: Front Physiol. 2019 Jul 12;10:870. doi: 10.3389/fphys.2019.00870 (PMC6639586; doi:10.3389/fphys.2019.00870)
Supplement: Supplementary file 1 [file Data_Sheet_1.PDF]

Supplementary Material

1 SUPPLEMENTARY FIGURES

Figure S1 shows relative changes of EMG amplitudes and frequencies after applying alternative methods to derive instantaneous amplitudes and frequencies from the raw EMG signals (this figure corresponds to Fig. 2 in the main text where EMG amplitudes and frequencies were obtained by the Hilbert transform/analytic signal approach). More specifically, after the initial high-pass filter with a cut-off at 10Hz was applied to the 2048Hz raw data, we have rectified the signal for instantaneous amplitude derivation, followed by a calculation of peak and root-mean-square (RMS) values in intervals of 30 sampling points. Figure S1(a,b) shows the averages of peak and RMS values for time intervals of one second.

Clearly, these more directly calculated amplitudes show a similar qualitative behavior compared to those derived by Hilbert transform (cp. Fig. 2(a)): we see a drop in EMG amplitudes during stop episodes for all subject groups and no drop during FOG episodes. Variations in the peak amplitudes (Fig. S1(a)) are similar to the Hilbert amplitudes. However, the drop is much weaker for the RMS amplitudes (Fig. S1(b)), in particular for the controls, so that only weak levels of significance can be achieved (note the modified vertical scale which does not include 0). A student's t-test yields $p < 0.01$ at t=0.5s for PD+FOG patients and at t=2.5s for EC subjects. In Fig. S1(c) the number of zero-crossing was counted in intervals of 0.125s, yielding an approximation for instantaneous frequencies at a sampling rate of 8Hz after multiplication by a factor of 4 (two zero-crossings per cycle and windows are 1/8-th of a second). These frequencies, shown in Fig. S1(c) exhibit nearly the same behavior as those derived by Hilbert transform and shown in Fig. 2(b) (also $p < 0.001$ for the deviations from 1 in most cases), although there are a bit stronger variations between muscles and subject groups for the direct frequencies.

Given the close resemblance of Fig. S1 and Fig. 2, we think that the instantaneous amplitudes and frequencies as derived by Hilbert transform and analytic signal approach are reliable and perhaps even more useful than deriving amplitudes an frequencies through more direct methods. In addition, the clearly visible stepping pattern in Fig. 1 (main text) indicates that even broad band Hilbert transform results in meaningful frequencies and amplitudes, which are related to the gait cycle. In contrast to rectification methods, Hilbert transform is a linear transformation and not susceptible to artificial frequency doubling.

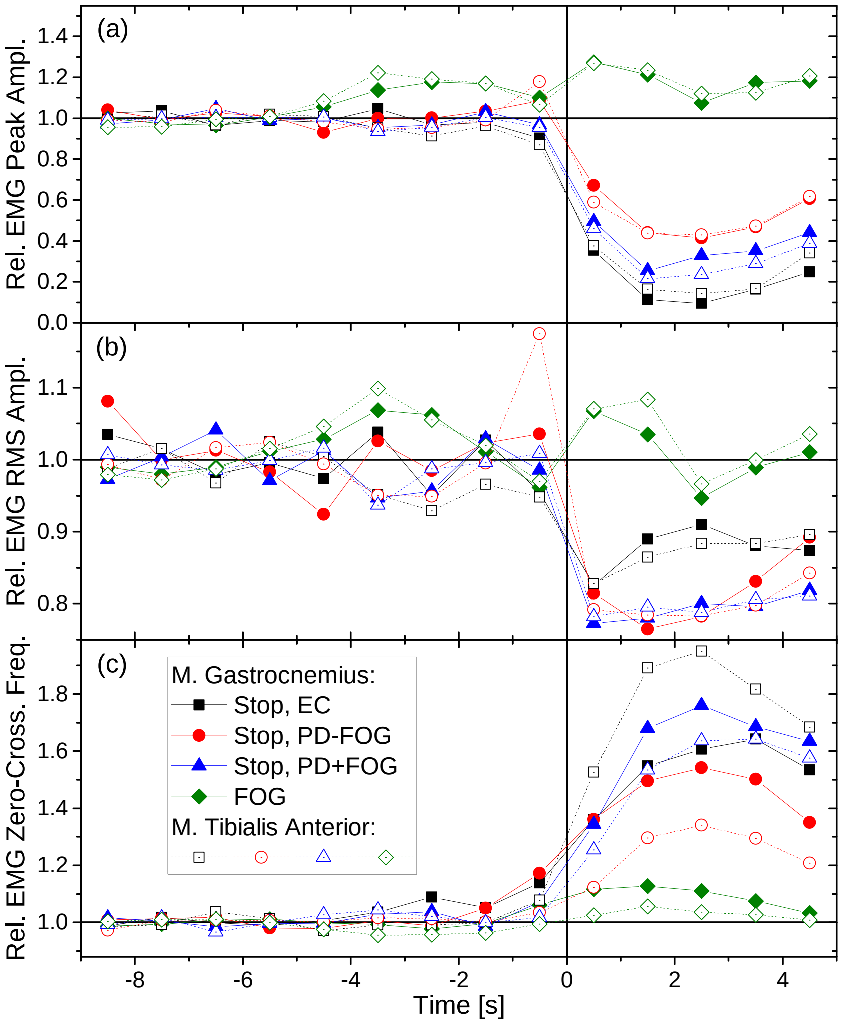

Figure S1. (Color online) Alternative methods to derive instantaneous amplitudes (a, b) and frequencies (c) from the raw EMG signals yield similar results as the Hilbert transfom/analystic signal approach. Compare this figure to Fig. 2 in the paper. For a detailed description of the applied methodologies, see text.

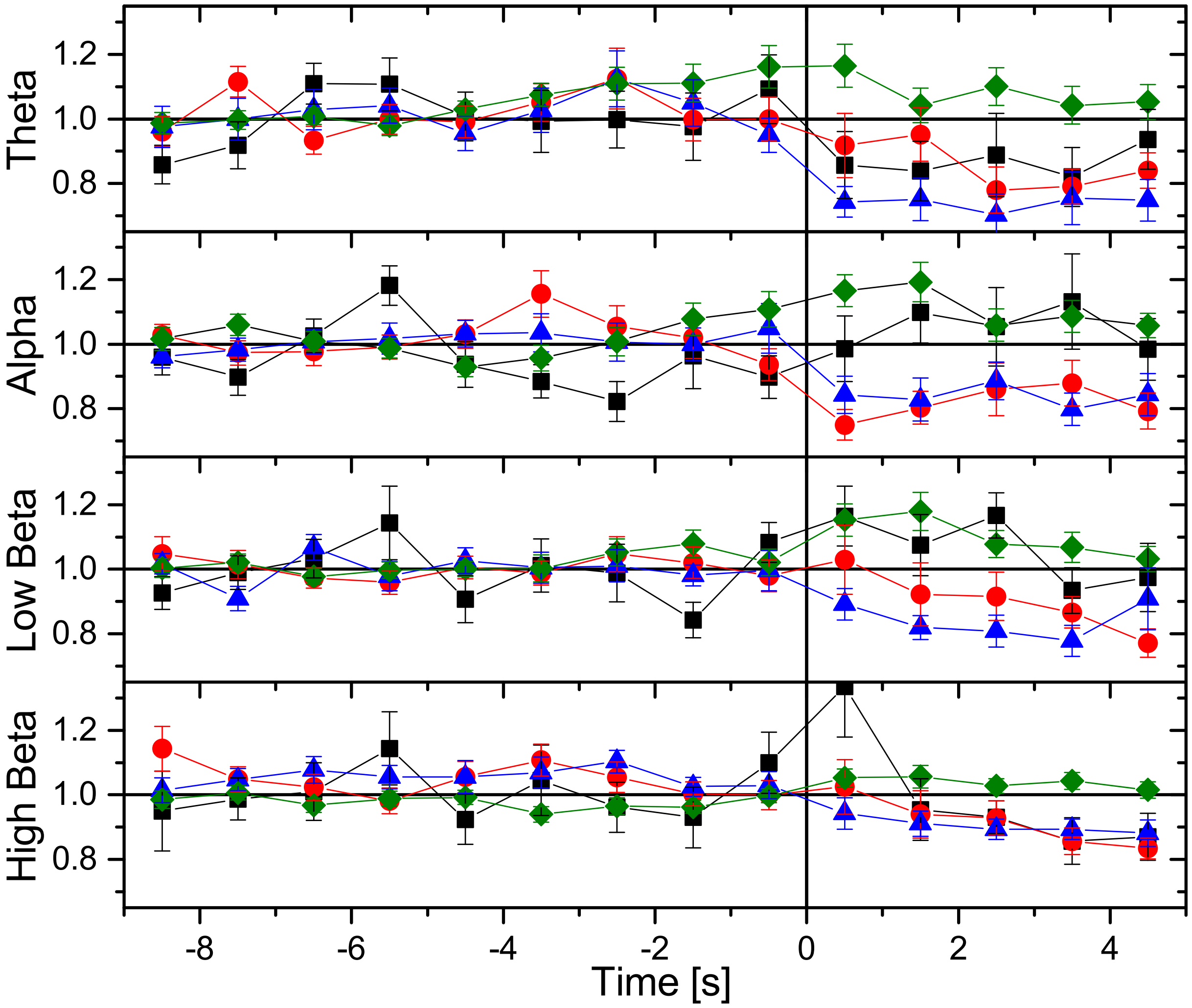

Figure S2. (Color online) Relative changes of EEG amplitudes during normal walking (time: -9s to 0s) followed by a commanded stop or FOG event (time: 0s to +5s) separately for the following EEG frequency bands: theta (4-7.5 Hz), alpha (7.5-13 Hz), low beta (13-21 Hz), and high beta (21-35 Hz). Data have been normalized to 1 for normal walking (time from -9 to -5 seconds). Error bars represent the standard error. This figure corresponds to Fig. 4 in the paper, where we present an average over all four bands.
